# Income-related inequalities in preventive and curative dental care use among working-age Japanese adults in urban areas: a cross-sectional study

**DOI:** 10.1186/1472-6831-14-117

**Published:** 2014-09-19

**Authors:** Keiko Murakami, Jun Aida, Takayoshi Ohkubo, Hideki Hashimoto

**Affiliations:** Department of Hygiene and Public Health, School of Medicine, Teikyo University, 2-11-1 Kaga, Itabashi-ku, Tokyo, 173-8605 Japan; Department of Health and Social Behavior, School of Public Health, The University of Tokyo, 7-3-1 Hongo, Bunkyo-ku, Tokyo, 113-0033 Japan; Department of International and Community Oral Health, Tohoku University Graduate School of Dentistry, 4-1 Seiryo-machi, Aoba-ku, Sendai, Miyagi, 980-8575 Japan

**Keywords:** Dental care, Dental insurance, Income, Inequality, Japan

## Abstract

**Background:**

Preventive dental care use remains relatively low in Japan, especially among working-age adults. Universal health insurance in Japan covers curative dental care with an out-of-pocket payment limit, though its coverage of preventive dental care is limited. The aim of this study was to test the hypothesis that income inequality in dental care use is found in preventive, but not curative dental care among working-age Japanese adults.

**Methods:**

A cross-sectional survey was conducted using a computer-assisted, self-administered format for community residents aged 25–50 years. In all, 4357 residents agreed to participate and complete the questionnaire (valid response rate: 31.3%). Preventive dental care use was measured according to whether the participant had visited a dentist or a dental hygienist during the past year for dental scaling or fluoride or orthodontic treatments. Curative dental care use was assessed by dental visits for other reasons. The main explanatory variable was equivalent household income. Logistic regression analyses with linear trend tests were conducted to determine whether there were significant income-related gradients with curative or preventive dental care use.

**Results:**

Among the respondents, 40.0% of men and 41.5% of women had used curative dental care in the past year; 24.1% of men and 34.1% of women had used preventive care. We found no significant income-related gradients of curative dental care among either men or women (*p* = 0.234 and *p* = 0.270, respectively). Significant income-related gradients of preventive care were observed among both men and women (*p* < 0.001 and *p* = 0.003, respectively). Among women, however, income-related differences were no longer significant (*p* = 0.126) after adjusting for education and other covariates. Compared with men with the lowest income, the highest-income group had a 1.79-fold significantly higher probability for using preventive dental care.

**Conclusions:**

The prevalence of preventive dental care use was lower than that of curative care. The results showed income-related inequality in preventive dental care use among men, though there were no significant income-related gradients of curative dental care use among either men or women. Educational attainment had a positive association with preventive dental care use only among women.

## Background

Many studies have consistently shown that an income-related inequality exists in dental care use [1–6]. Affluent people have better access to dental care than the less affluent despite the fact that dental care needs are more severe among the less affluent [7–9]. The magnitudes of inequalities were larger than those for other types of health care, which is often attributed to the fact that dental insurance is not provided publicly and has to be paid for either out-of-pocket or through private insurance coverage in many developed countries. Japan is one of the exceptions. Since the start of its universal health insurance coverage in 1961, the system has covered inpatient, outpatient, and dental care [10]. Unlike medical care, however, dental care allows extra charges out of insurance coverage, and it covers preventive dental care only for limited conditions [11].

There are extensive prevention possibilities, and prevention can actually save resources especially in case of dental care [12]. Dental care use in Japan has traditionally been treatment-oriented, and the prevalence of preventive dental care use remains relatively low especially among working-age adults; for example, the percentages of those who reported having dental check-ups in the past year were 29.4% of people in their twenties, 32.2% of those in their thirties or forties, while that was 41.4% of those in their sixties [13]. The concept of prevention has become widespread in recent decades, and there has been improvement in oral health behaviors including in Japan [14, 15]. However, such spreading of preventive behaviors is often accompanied by inequalities across socioeconomic positions, due to differential uptake of new information and skills. In other words, increased preventive behavior often results in more disadvantage among lower social groups than higher ones [16]. In addition to individual education for preventive behavior, a system to facilitate access to preventive care is imperative to drive oral health improvement on a population level. In this regards, public insurance coverage for preventive care would narrow the gap.

As mentioned above, some forms of preventive dental care are not covered by insurance in Japan, so we used the situation to test a hypothesis that the income gap in service use is found in preventive dental care but not in curative dental care, which is covered by insurance. There might be significant value to examine how the different coverage between curative and preventive care would affect income-related inequalities in dental care use in one country.

## Methods

### Data

The data were derived from the Japanese Study of Stratification, Health, Income, and Neighborhood (J-SHINE). The J-SHINE survey was conducted between October 2010 and February 2011 in four municipalities in and around the Tokyo metropolitan area. The survey participants were community residents aged 25–50 years who were randomly selected from the residential registry. The sampling strategy for this survey is described in detail elsewhere [17]. Of 13920 residents who were randomly selected, after excluding those individuals who had moved, were absent, or could not be located, survey staffs could have contact with 8408 residents (contact success rate: 60.4%). Those individuals who agreed to complete the survey were asked to complete the questionnaire in a computer-assisted, self-administered format unless the participants requested a face-to-face interview. Among them, a total of 4357 residents agreed to participate and complete the questionnaire (response rate: 31.3%; cooperation rate: 51.8%). From the responses received, we used an analytic sample of 3083 individuals who did not have missing values for household income, age, gender, self-assessed oral health, marital status, educational attainment, work status, and dental care use (22.1% of the originally selected sample). Because this attrition was mainly due to non-response on income, we applied a multiple imputation of income and obtained similar results. We therefore present only the results without imputation of income. The secondary use of the data was approved by the 2011 data management committee of the J-SHINE research group, with personally identifiable information deleted to ensure confidentiality.

### Measures

Curative dental care use in the past year was measured through self-reporting in response to the question, “In the past year, have you been seen by a dentist or a dental hygienist? Exclude use for dental scaling and fluoride and orthodontic treatments.” Preventive dental care use in the past year was measured through self-report responses to the question, “In the past year, have you been seen by a dentist or a dental hygienist for dental scaling or fluoride or orthodontic treatments?”

Each respondent was asked to select his/her total annual household income from among 15 categories: <250, 250–500, 500–750, 750–1000, 1000–1250, 1250–1500, 1500–2000, 2000–3000, 3000–4000, 4000–5000, 5000–7500, 7500–10000, 10000–15000, 15000–20000, and >20000 thousand Japanese yen. It was made clear that household income included stock dividends, extra income, and additional income. The equivalent household incomes were derived from adjustment for household size using the OECD-modified equivalence scale [18].

The respondents were sorted into three categories of educational attainment: high school or less (elementary, junior high, or senior high school), two-year college or special training school, and university or higher (university or graduate school). Self-assessed oral health was determined by answers to the question, “How would you describe the health of your teeth and gums? Would you say it is excellent, very good, good, fair, or poor?” [19, 20]. Since the number of respondents in the “poor” stratum category was small, “poor” and “fair” categories were combined into one “poor/fair” category for analysis purposes.

### Statistical analysis

Differences in the proportions of selected characteristics of respondents by gender were assessed by chi-square test. Multiple logistic regression analyses were conducted to examine the association of household income with curative and preventive dental care use. Crude odds ratios, and odds ratios adjusted for other social and dental variables (age, marital status, educational attainment, work status, city of residence, and self-assessed oral health), and their 95% confidence intervals, were calculated. A trend test was also conducted to test the linear trend between household income and dental care use. Some previous studies found gender differences in the association between socioeconomic status and health outcomes. For example, one study on elderly Japanese people identified gender differences among the factors related to dental care access, including income indicators [21]. To test the significance of distinct associations between socioeconomic status (i.e., income and educational attainment) and dental care use according to gender, the interaction term between each socioeconomic indicator and gender was entered into the model. The results showed no significant interaction between socioeconomic status and gender for curative dental care use (income *p* = 0.816 and educational attainment *p* = 0.397, respectively). For preventive dental care, the interaction term between educational attainment and gender was statistically significant (*p* = 0.022), but no significant interaction between income and gender was found (*p* = 0.500). On the basis of these results, we present only the results of the analyses conducted separately for men and women.

Statistical analysis was conducted using STATA (release 12; Windows version). All analyses were two-tailed, and *p*-values less than 0.05 were considered statistically significant.

## Results

Table 1 presents the sociodemographic characteristics of the respondents by gender. Men were more likely to be highly educated, be currently working, have higher household income, and were less likely to be married by formal or common law than women.

**Table 1:** **Characteristics of respondents by gender: Japanese Study of Stratification, Health, Income, and Neighborhood (J-SHINE)**

		Men (n = 1494)		Women (n = 1589)		***p***-value*
Age, no. (%)						0.625
	25-34 years	478	(32.0)	534	(33.6)	
	35-44 years	676	(45.3)	698	(43.9)	
	45-50 years	340	(22.8)	357	(22.5)	
Marital status, no. (%)						< 0.001
	Married/Common-law	1118	(74.8)	1285	(80.9)	
	Others	376	(25.2)	304	(19.1)	
Educational attainment, no. (%)						< 0.001
	≤ High school	330	(22.1)	334	(21.0)	
	College	318	(21.3)	715	(45.0)	
	≥ University	846	(56.6)	540	(34.0)	
Work status, no. (%)						< 0.001
	Working	1431	(95.8)	1006	(63.3)	
	Not working	63	(4.2)	583	(36.7)	
City of residence, no. (%)						0.849
	City 1	290	(19.4)	296	(18.6)	
	City 2	362	(24.2)	402	(25.3)	
	City 3	425	(28.5)	459	(28.9)	
	City 4	417	(27.9)	432	(27.2)	
Self-assessed oral health, no. (%)						< 0.001
	Fair/Poor	439	(29.4)	373	(23.5)	
	Good	486	(32.5)	629	(39.6)	
	Very good	356	(23.8)	396	(24.9)	
	Excellent	213	(14.3)	191	(12.0)	
Income, thousand JPY^†^ (/year), median (interquartile range)		3500.0	(2361.1)	3472.2	(2166.7)	< 0.001

*p-value for differences by chi-square test. Income difference was tested by *t*-test.

^†^JPY: Japanese Yen.

Table 2 shows prevalence, the odds ratios (ORs) and 95% confidence intervals (CIs) for curative dental care use by gender. The percentages of those who used curative dental care during the past year were 40.0% and 41.5% among men and women, respectively. No significant income-related gradients of curative care were found among either men or women (trend test *p* = 0.234 and *p* = 0.270, respectively). In addition, none of age, marital status, educational attainment, work status, or city of residence was significantly associated with curative care. The likelihood of curative dental care use was higher among those who assessed their own oral health as worse than among those as better.

**Table 2:** **Prevalence, odds ratio (OR) and 95% confidence interval (CI) for curative dental care use**

			Men (n = 1494)					Women (n = 1589)			
		%	Crude OR (95% CI)		Adjusted OR* (95% CI)		%	Crude OR (95% CI)		Adjusted OR* (95% CI)	
Total		40.0					41.5				
Income quartiles			*p* ^†^ = 0.440		*p* ^†^ = 0.234			*p* ^†^ = 0.332		*p* ^†^ = 0.270	
	1 (lowest)	37.5	1.00		1.00		42.9	1.00		1.00	
	2	42.1	1.21	(0.91-1.61)	1.29	(0.96-1.74)	35.8	0.74	(0.57-0.98)	0.75	(0.57-0.99)
	3	38.9	1.06	(0.79-1.43)	1.12	(0.82-1.54)	46.2	1.14	(0.85-1.53)	1.17	(0.86-1.58)
	4 (highest)	41.4	1.18	(0.88-1.57)	1.28	(0.94-1.75)	43.0	1.00	(0.76-1.33)	1.03	(0.76-1.40)
Age											
	25-34 years	41.6	1.00		1.00		39.3	1.00		1.00	
	35-44 years	35.8	0.78	(0.61-0.99)	0.72	(0.56-0.93)	41.8	1.11	(0.88-1.40)	1.04	(0.82-1.32)
	45-50 years	45.9	1.19	(0.90-1.57)	1.05	(0.78-1.42)	44.3	1.22	(0.93-1.61)	1.08	(0.81-1.43)
Marital status											
	Married/Common-law	41.1	1.00		1.00		42.3	1.00		1.00	
	Others	36.7	0.83	(0.65-1.06)	0.84	(0.65-1.09)	38.2	0.84	(0.65-1.09)	0.81	(0.61-1.07)
Educational attainment											
	≤ High school	40.3	1.00		1.00		42.8	1.00		1.00	
	College	40.3	1.00	(0.73-1.37)	1.04	(0.75-1.43)	42.4	0.98	(0.76-1.28)	1.01	(0.77-1.32)
	≥ University	39.7	0.98	(0.75-1.26)	1.06	(0.80-1.39)	39.6	0.88	(0.66-1.16)	0.91	(0.67-1.22)
Work status											
	Working	40.2	1.00		1.00		42.9	1.00		1.00	
	Not working	34.9	0.80	(0.47-1.36)	0.90	(0.52-1.58)	39.1	0.85	(0.69-1.05)	0.85	(0.68-1.06)
City of residence											
	City 1	42.4	1.00		1.00		45.6	1.00		1.00	
	City 2	38.4	0.85	(0.62-1.16)	0.86	(0.62-1.19)	42.8	0.89	(0.66-1.21)	0.92	(0.67-1.25)
	City 3	40.2	0.91	(0.68-1.24)	0.92	(0.67-1.25)	42.3	0.87	(0.65-1.17)	0.92	(0.68-1.25)
	City 4	39.3	0.88	(0.65-1.19)	0.89	(0.65-1.21)	36.8	0.69	(0.51-0.94)	0.74	(0.54-1.00)
Self-assessed oral health											
	Fair/Poor	50.1	1.00		1.00		48.3	1.00		1.00	
	Good	37.2	0.59	(0.45-0.77)	0.59	(0.45-0.77)	42.1	0.78	(0.60-1.01)	0.79	(0.61-1.02)
	Very good	37.1	0.59	(0.44-0.78)	0.57	(0.43-0.77)	38.1	0.66	(0.50-0.88)	0.69	(0.52-0.93)
	Excellent	30.1	0.43	(0.30-0.61)	0.42	(0.29-0.59)	33.5	0.54	(0.38-0.78)	0.56	(0.39-0.81)

*Adjusted for all other variables shown in the table.

^†^ *p*-value of trend test.

Table 3 presents prevalence, the ORs and 95% CIs for preventive dental care use by gender. More women than men had used preventive dental care during the past year (34.1% compared with 24.1%). Among men, there was a significant income-related gradient of preventive care use, even after adjusting for covariates (trend test *p* = 0.001). Compared to men with the lowest income, only those in the highest income group had significantly higher probability of using preventive dental care (*p* = 0.003). Women in higher household income groups were also more likely to use preventive dental care (trend test *p* = 0.003). However, after adjusting for education and other covariates, the income-related difference in preventive care substantially decreased and was no longer significant (trend test *p* = 0.126). We found a significant association between educational attainment and preventive care among women, although this was not seen for men. The likelihood of preventive dental care use was higher among those who assessed their own oral health as better than among those as worse.

**Table 3:** **Prevalence, odds ratio (OR) and 95% confidence interval (CI) for preventive dental care use**

			Men (n = 1494)					Women (n = 1589)			
		%	Crude OR (95% CI)		Adjusted OR* (95% CI)		%	Crude OR (95% CI)		Adjusted OR* (95% CI)	
Total		24.1					34.1				
Income quartiles			*p* ^†^ < 0.001		*p* ^†^ = 0.001			*p* ^†^ = 0.003		*p* ^†^ = 0.126	
	1 (lowest)	19.8	1.00		1.00		29.2	1.00		1.00	
	2	20.9	1.08	(0.76-1.52)	1.07	(0.75-1.54)	33.1	1.20	(0.90-1.60)	1.11	(0.83-1.50)
	3	25.8	1.41	(1.00-2.00)	1.42	(0.99-2.05)	36.3	1.38	(1.01-1.88)	1.24	(0.90-1.71)
	4 (highest)	30.5	1.79	(1.28-2.49)	1.72	(1.20-2.46)	38.6	1.53	(1.14-2.05)	1.26	(0.91-1.73)
Age											
	25-34 years	23.4	1.00		1.00		34.6	1.00		1.00	
	35-44 years	21.5	0.89	(0.67-1.18)	0.87	(0.65-1.16)	32.2	0.90	(0.71-1.14)	0.97	(0.75-1.24)
	45-50 years	30.3	1.42	(1.04-1.94)	1.34	(0.96-1.88)	37.0	1.11	(0.84-1.46)	1.18	(0.88-1.59)
Marital status											
	Married/Common-law	24.1	1.00		1.00		34.5	1.00		1.00	
	Others	24.2	1.01	(0.77-1.32)	1.00	(0.74-1.35)	32.6	0.92	(0.70-1.20)	0.93	(0.70-1.25)
Educational attainment											
	≤ High school	23.9	1.00		1.00		25.2	1.00		1.00	
	College	22.0	0.90	(0.62-1.29)	0.92	(0.63-1.34)	35.2	1.62	(1.21-2.17)	1.55	(1.15-2.08)
	≥ University	24.9	1.06	(0.78-1.42)	0.89	(0.65-1.21)	38.2	1.84	(1.36-2.48)	1.66	(1.20-2.29)
Work status											
	Working	23.9	1.00		1.00		33.5	1.00		1.00	
	Not working	28.6	1.27	(0.73-2.23)	1.43	(0.79-2.59)	35.2	1.08	(0.87-1.33)	1.11	(0.88-1.40)
City of residence											
	City 1	20.7	1.00		1.00		35.1	1.00		1.00	
	City 2	24.6	1.25	(0.86-1.81)	1.14	(0.78-1.67)	37.6	1.11	(0.81-1.52)	0.96	(0.69-1.33)
	City 3	27.8	1.47	(1.03-2.10)	1.43	(1.00-2.05)	34.2	0.96	(0.71-1.30)	0.86	(0.63-1.18)
	City 4	22.3	1.10	(0.76-1.59)	1.08	(0.75-1.57)	30.1	0.79	(0.58-1.09)	0.72	(0.52-0.99)
Self-assessed oral health											
	Fair/Poor	21.2	1.00		1.00		31.9	1.00		1.00	
	Good	24.9	1.23	(0.91-1.68)	1.25	(0.91-1.70)	29.7	0.90	(0.68-1.19)	0.91	(0.69-1.21)
	Very good	22.5	1.08	(0.77-1.51)	1.04	(0.74-1.48)	39.9	1.42	(1.05-1.91)	1.39	(1.03-1.89)
	Excellent	31.0	1.67	(1.15-2.42)	1.62	(1.10-2.36)	40.8	1.47	(1.03-2.11)	1.41	(0.98-2.04)

*Adjusted for all other variables shown in the table.

^†^ *p*-value of trend test.

## Discussion

This study explored income-related inequality in curative and preventive dental care use among working-age Japanese men and women. The prevalence of preventive dental care use was lower than that of curative one. We found no significant income-related gradients of curative dental care among either men or women. Significant income-related gradients in preventive dental care were observed for both men and women. Among women, however, the income-related difference was no longer significant after adjusting for education and other covariates. Educational attainment was positively associated with preventive dental care use only for women.

The present study found income-related inequalities in preventive dental care use among men, but no significant inequalities in curative one, as expected from the insurance coverage status of the two types of dental care. It is noteworthy that use of curative dental care, which is mostly covered by public insurance in Japan, showed no income-related inequality. In Canada, the income-related inequality in frequent preventive dental care was larger than that measured in total dental care [22]. In European countries, analyses of the data from a representative sample aged 50 and over have also shown that a huge proportion of income-related inequalities in dental care use was attributable to inequalities in preventive care, but not to differential use of operative care alone [23]. The present findings are generally in agreement with these previous studies.

The results of gender differences in income-related inequalities are probably derived from the argument that income may not reflect a woman’s position in the social hierarchy [24]. Several studies have demonstrated that social inequalities in health are greater when assessing the most dominant social position in the household than a woman’s own social position and that this applies in Japan [25]. A previous study using a representative sample in Japan showed that household income may exert a direct effect on psychological distress among men, but not among women [26].

One study on elderly Japanese people found that there was a tendency only for men with low equivalent income not to visit dental clinics regularly [21]. In light of these results, factors other than income may affect preventive dental care among women. Women tend to use preventive health care services more frequently than men, partly because women report more interest in health [27]. Dental scaling, which accounts for a considerable proportion of preventive dental care, is of mainly two types: one is aftercare treatment for periodontal disease; the other is purely preventive care, irrespective of periodontal disease. The present study showed that the likelihood of preventive dental care use was higher among women than among men, which implies that women tend to use preventive dental care irrespective of periodontal disease. No gender difference in the likelihood of using curative dental care was observed. Such preventive care may be associated with knowledge of or consciousness related to dental health [12, 15]; therefore, our results indicate that educational attainment was associated with preventive care only among women.

Because the data were cross-sectional in nature, the survey assessed dental care use in the past year, whereas oral health was assessed at the time of the survey only. There were therefore temporal inconsistencies between actual dental care use and oral health status. Previous studies have shown that self-assessed oral health was positively associated with dental care use; this result confirmed that self-assessed oral health status probably represents the outcome of dental care rather than the need for it [22, 28]. In the present study, self-assessed oral health showed opposite gradients for the two types of dental care: those who assessed their own oral health as better were less likely to use curative, but more likely to use preventive dental care than others, which was similar to the results of a previous study in the United States [29]. The result of preventive care is likely due to the reverse causality.

These findings have some implications for dental care policy. Universal coverage seems to function effectively for curative dental care, while further steps to promote preventive dental care should be needed, particularly considering the impact of income on its use. One promising strategy which does not rely on individual choice may be to include oral health as part of the mandatory annual general health check in Japan. The Japanese health care system has the unique characteristic of including a mandatory annual health checkup for all, but a dental checkup is not included in most settings. In a randomized controlled trial in the UK, the inclusion of a dental checklist within the preventive health checkup for elderly patients together with help arranging a dental appointment has shown that the offer of dental care was taken up most readily by those with current oral health problems, pain, and no regular dentist [30]. Although economic evaluation in the field of dentistry is undeniably lacking [31], the introduction and cost-effectiveness of these promising strategies may be important.

Some limitations of the present study should be considered. First, the response rate was low. This was largely due to the low contact success rate (60.4%). Survey staffs attempted to contact potential participants at least six times, but over one-third of them could not be reached. Several previous studies have examined nonparticipation in surveys by linking it with register information, and found that compared with respondents, non-respondents have low socioeconomic status such as income and educational attainment [32, 33]. If such a non-response bias existed in the present study, socioeconomic inequalities in dental care use would be underestimated. However, the sample obtained was fairly comparable with the target population in terms of age and sex distribution, and percentages of graduates of high school or less in Census 2010 [17]. Second, the sampled municipalities were all located in urban areas, where the demographic structure and access to dental care may be different from rural areas. The findings should therefore be generalized only with caution. However, a previous study revealed that there is an oversupply of dentists in Japan, which has reached a level such that the nationwide distribution of dentists has become relatively homogenous [34]. Third, the measurement of dental care use remains controversial. The present study examined curative and preventive dental care separately, which is its strength. However, it could be difficult to distinguish use for exclusively preventive purposes from that for both curative and preventive purposes. Unfortunately, the definition of dental care (both preventive and curative) differs among studies or countries [22, 29]. To our knowledge, previous studies have not defined or examined preventive dental care in Japan in detail. Because dental scaling, fluoride, and orthodontic treatments are a better proxy for placing a high priority on good oral health than other dental care (such as fillings, crowns, and root canals), we believe that the definition of preventive dental care in the present study is suitable at the moment. In addition, the quality of dental care used could not be examined because of data limitations. Finally, the J-SHINE data were based on self-reports. Income data could be subject to bias due to under-reporting, over-reporting, and a non-negligible number of missing values. More sophisticated methods for eliciting accurate income information (e.g., in-person interviews) have been developed, but of course these come at a cost of having to devote more space and time to collect these data [35]. In addition, even these methods could not eliminate reporting biases completely. Also, income data in this study was divided into discrete numbers of categories, which could lead to measurement error. In general, people in lower income groups are less likely to report their income status, which could lead to bias. Compared with nationally representative data, such as the Comprehensive Survey of the Living Conditions of People on Health and Welfare in Japan, 2010 [36], the mean equivalent income in the present study was higher than national per capita income. This result might have led to underestimation of the association. However, as mentioned above, our analysis using a multiple imputation of income showed similar results, making it less likely that non-response on income substantially affected the results.

## Conclusions

Among working-age Japanese men and women in urban areas, the prevalence of preventive dental care use was lower than that of curative care. The results showed income-related inequality in preventive dental care use among men, though there were no significant income-related gradients of curative dental care use among either men or women. In addition, educational attainment had a positive association with preventive dental care use only among women.
